# High-Contrast Fluorescence Microscopy for a Biomolecular Analysis Based on Polarization Techniques Using an Optical Interference Mirror Slide

**DOI:** 10.3390/bios4040513

**Published:** 2014-12-15

**Authors:** Mitsuru Yasuda, Takuo Akimoto

**Affiliations:** School of Bioscience and Biotechnology, Tokyo University of Technology, 1404-1 Katakura, Hachioji, Tokyo 192-0982, Japan; E-Mail: m.yasuda@kwansei.ac.jp

**Keywords:** fluorescence microscopy, fluorescence enhancement, biochip

## Abstract

Fluorescence microscopy with an improved contrast for fluorescence images is developed using an optical interference mirror (OIM) slide, which can enhance the fluorescence from a fluorophore as a result of the double interference of the excitation light and emission light. To improve the contrast of a fluorescence image using an OIM slide, a linearly-polarized excitation light was employed, and the fluorescence emission polarized perpendicular to the polarization of the excitation light was detected. The image contrast with this optical system was improved 110-fold for rhodamine B spotted on the OIM, in comparison with a glass slide using a general fluorescence microscopy optical system. Moreover, a 24-fold improvement of the image contrast was achieved for the detection of Cy3-labeled streptavidin bound to immobilize biotin.

## 1. Introduction

Enhancing the fluorescence signal is a crucial technique for chip-based protein and DNA analysis, as well as for microscopic biomolecules and cell imaging. Increasing the exposure time of the charge-coupled device (CCD) detector is a simple approach to obtain a bright fluorescence image [1]. However, a shorter exposure time is frequently required to obtain continuous imaging of a moving target. To obtain a bright image with a short exposure time, enhancing the fluorescence signal over a short exposure time is preferred. 

One approach for addressing this issue includes the use of a substrate with a micro-/nano-structured surface. For example, plasmonic chips are known as typical fluorescence enhancement substrates based on surface plasmon resonance [2] and localized surface plasmon resonance [3]. Substrates for enhancing fluorescence using pillar [4], porous [5], waveguide [6], photonic crystal [7] and metal film [8] structures have also been developed. Fluorescence enhancements of several-fold to as high as 50-fold can be achieved with these substrates in comparison with standard substrates.

An optical interference mirror (OIM) slide consisting of a transparent dielectric thin-film on a flat Ag substrate has been reported to enhance fluorescence emissions [9]. The fluorescence signal using an OIM slide can be enhanced by greater than 100-fold relative to that using a bare glass slide [9,10,11,12]. This enhancement is mainly accounted for by the optical interference of both the excitation light and the fluorescence emission in the dielectric layer [13].

The fluorescence enhancement obtained by an OIM slide has been applied to acquire two-dimensional fluorescence images for biomolecular analysis [14], environmental monitoring [15] and cell imaging [16,17]. On the other hand, the contrast of the fluorescence images obtained using an OIM slide is not sufficient because of the increase in background noise owing to strong back scattering of the excitation light on the Ag surface [13]. Therefore, a reduction of the back-scattered light is required to improve the contrast of the resulting fluorescence images. To this end, we have focused on the polarization of the back-scattered light and the fluorescence emission from the OIM slide.

The enhanced fluorescence owing to the use of an OIM slide is known to be dependent on the polarization of the excitation light, and it has been established that TE (transverse electric)-polarized excitation light enhances the fluorescence, whereas TM (transverse magnetic)-polarized excitation light does not. Consequently, it has been found that the electric field of the excitation light oscillating parallel to the OIM surface can enhance the fluorescence [18]. Use of this polarization dependence of the fluorescence enhancement has provided for the development of a high-contrast fluorescence imaging system in which a TE-polarized excitation light source was used to enhance the fluorescence emission, and the TM-component of the fluorescence emission was detected to eliminate the back-scattered excitation light [19].

In this study, we applied a high contrast fluorescence imaging technique using an OIM slide to a commercial fluorescence microscope and obtained improved contrast fluorescence images for biomolecular analysis. Since the incident angle of the excitation light was nearly zero degrees to the OIM slide for the fluorescence microscope, the electric field of the excitation light oscillates parallel to the OIM surface. Therefore, the enhancement of the fluorescence can be maximized by the fluorescence microscope. For the present study, the polarizations of the back-scattered excitation light and the fluorescence emission were first investigated while employing a linearly-polarized excitation light. The image contrast for a fluorophore spotted on the OIM slide was then estimated, and a fluorophore-labeled biomolecule was finally detected.

## 2. Experimental Section 

### 2.1. Reagents and Proteins

Rhodamine B and (3-aminopropyl)trimethoxysilane were purchased from Sigma-Aldrich Co. (St. Louis, MO, USA). A sulfo-NHS biotin derivative (biotin N-hydroxy-sulfosuccinimide ester) was obtained from Dojindo Laboratories (Kumamoto, Japan). Cy3-labeled streptavidin was purchased from GE Healthcare U.K. Ltd. (Buckinghamshire, England). Bovine serum albumin (BSA) and 2-amino-2-hydroxymethyl-1,3-propanediol (Tris) were obtained from Wako Pure Chemical Industries, Ltd. (Osaka, Japan). For analysis, 10 mM Tris-HCl buffer (pH 7.4) was prepared.

### 2.2. Fabrication of an Optical Interference Mirror Slide

According to the procedure described previously [13,18], the OIM slide was fabricated using sputtering equipment (CFS-4ES, Shibaura). Briefly, microscope glass slides were first sonicated in ethanol and then H_2_O for 1 h each and then rinsed with H_2_O. After drying in an oven at 100 °C, Cr, as an adhesive, Ag, as a mirror, and Al_2_O_3_, as an optical interference layer, were sequentially sputtered on the cleaned glass slides. The thicknesses of the Cr and Ag layers were 15 nm and 1 µm, respectively.

### 2.3. Preparation of Rhodamine B

Two types of rhodamine B solutions were prepared, as reported previously [19]. To investigate the polarization of the fluorescence emission from the OIM slide, 30 µg/mL of rhodamine B solution diluted with ethanol were spin-coated onto the Al_2_O_3_ surface of the OIM slide. For the estimation of the image contrast, 100 ng/mL of rhodamine B solution dissolved in H_2_O were prepared. Ten nanoliters of the prepared rhodamine B solution were spotted onto the Al_2_O_3_ surface of the OIM slide using a spotter (BioChip Arrayer, PerkinElmer) and then dried at room temperature.

### 2.4. Detection of Streptavidin

For detection, Cy3-labeled streptavidin was bonded to biotin immobilized on the OIM slide by amide binding. The OIM slide was first aminated by incubating in a solution of 1% (3-aminopropyl)trimethoxysilane in H_2_O for 1.5 h. The OIM slide was washed in H_2_O for 10 min followed by drying in an oven for 10 min at 110 °C.

To immobilize biotin via amide binding, 200 µg/mL of the sulfo-NHS biotin derivative dissolved in H_2_O were spotted onto the aminated Al_2_O_3_ surface of the OIM slide using a spotter (BioChip Arrayer, PerkinElmer). The volume of the spotted solution was 10 nL. The OIM slide was then immersed in water containing 10 mg/mL of BSA as a blocking procedure. The OIM slide was washed in H_2_O for 30 min, followed by drying at room temperature.

For detecting Cy3-labeled streptavidin, 10 nL of a Cy3-labeled streptavidin solution with a concentration of 1.0 µg/mL dissolved in H_2_O were spotted onto the biotin spot area of the OIM slide, and the OIM slide was immersed in a Tris-HCl buffer for 30 min to react the Cy3-labeled streptavidin with the immobilized biotin. The OIM slides were finally washed in the Tris-HCl buffer for 30 min and then dried at room temperature.

### 2.5. Optical System

**Figure 1:**
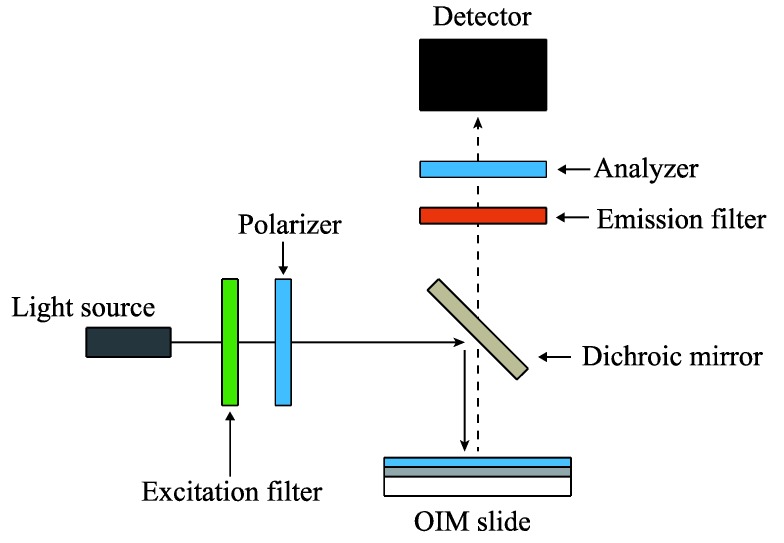
Optical arrangement of the microscope. The polarizer was inserted to polarize the excitation light. The other polarizer, denoted as “Analyzer”, was inserted to measure the polarization of the back scattered excitation light and fluorescence emission. OIM, optical interference mirror.

A commercially available epi-fluorescence microscope (BX50, Olympus) was used as an instrument for detecting the fluorescence emission. The microscope was equipped with two polarizers. One was used to polarize the excitation light. The other was used to polarize the emission light and the back-scattered excitation light, denoted as “Analyzer” in Figure 1. Excitation light of a 545-nm wavelength extracted from a mercury lamp using a band pass filter was focused on the OIM with a 10× objective lens. The fluorescence from rhodamine B spin-coated on the OIM was detected with a cooled CCD camera (C4742-8-12AG, Hamamatsu Photonics) via a 610-nm band pass filter.

The analyzer inserted between the emission filter and the CCD camera was used to analyze the polarization of the back-scattered excitation light and the fluorescence emission from the OIM. To examine the polarization of the back-scattered excitation light from the OIM, the back-scattered light was detected without the emission filter and with an OIM not coated with rhodamine B. All image data were analyzed using image analysis software (Image-Pro Plus, Media Cybernetics).

### 2.6. Design of the Al_2_O_3_ Thickness

The thickness *d* of the Al_2_O_3_ layer on the OIM slide for maximum fluorescence enhancement varies according to the incident angle of the excitation light [18]. Because the numerical aperture of the objective lens used in this research was 0.3, the maximum incident angle of the excitation light to the OIM was 17.5°. Assuming the refractive index of Al_2_O_3_ to be 1.62 [20], *d* was estimated to be 90 nm from basic optical interference theory [13,14].

## 3. Results and Discussion

### 3.1. Polarizations of the Back-Scattered Excitation Light and Fluorescence Emission

**Figure 2:**
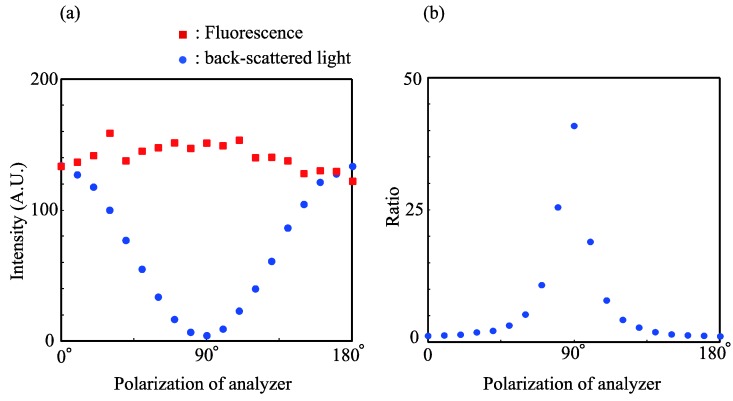
(**a**) Polarizations of the back-scattered light and the fluorescence emission from the OIM slide. The solid curves are drawn as guides. (**b**) The ratio between the fluorescence and the back scattered light. The solid curve is drawn as guides.

The polarization of the back-scattered excitation light from the OIM slide was firstly investigated. In this investigation, a linearly-polarized excitation light at an arbitrary angle of polarization was incident on the bare OIM slide, because the distinction between the TE and TM polarizations of the excitation light can be ignored for the fluorescence microscope used in this investigation. Figure 2a shows the polarization of the back-scattered light. The back-scattered light exhibited a minimum for polarization normal to the excitation light and exhibited a maximum for polarization parallel to the excitation light. This indicates that the back-scattered light maintains the polarization of the incident excitation light.

Thereafter, the polarization of the fluorescence emission was investigated using the linearly-polarized excitation light and a rhodamine B-coated OIM slide, and the results are shown in Figure 2a. From Figure 2a, it can be observed that the fluorescence intensity was nearly constant, indicating that the fluorescence emission was unpolarized, even though the incident excitation light was polarized.

Although the fluorescence from a fluorophore excited by polarized light is known to be polarized, the fluorescence emission observed in the present work was unpolarized. The observed depolarization of the fluorescence emission is expected to have been caused by the surface roughness of the OIM slide. The fluorescence from the rhodamine B was scattered at the rough surface, which results in the random rotation of the polarization axis of the fluorescence emission [19].

We had investigated the polarization of the back-scattered light and fluorescence emission using TE-polarized excitation light with 20, 60 and 75 degrees of incident angles. The fluorescence emission was found to be unpolarized, and the back-scattered excitation light was TE-polarized. Moreover, the fluorescence emission was unpolarized, and the back-scattered excitation light was TM-polarized, when TM-polarized excitation light was employed. These observations indicated that the fluorescence emission was always unpolarized regardless of the incident angle and the polarization of the excitation light [19]. The reason for the depolarization of the fluorescence emission is under investigation. However, it is not yet completely understood.

On the basis of the difference of polarization between the back-scattered light and the fluorescence emission, an optimal optical system that can provide the maximum contrast for the fluorescence image was designed. For this system, the ratio of the fluorescence emission to the background light was calculated and is presented in Figure 2b. From Figure 2b, the condition where the analyzer is polarized perpendicular to the polarization of the excitation light is found to provide maximum image contrast.

A configuration where the analyzer is polarized perpendicular to the polarization of the excitation light is commonly referred to as a crossed Nicols configuration, and the optical system is therefore referred to as a crossed Nicols optical system. A configuration without an analyzer and unpolarized excitation light is referred to as a general optical system.

### 3.2. Evaluation of the Image Contrast for Rhodamine B

**Figure 3:**
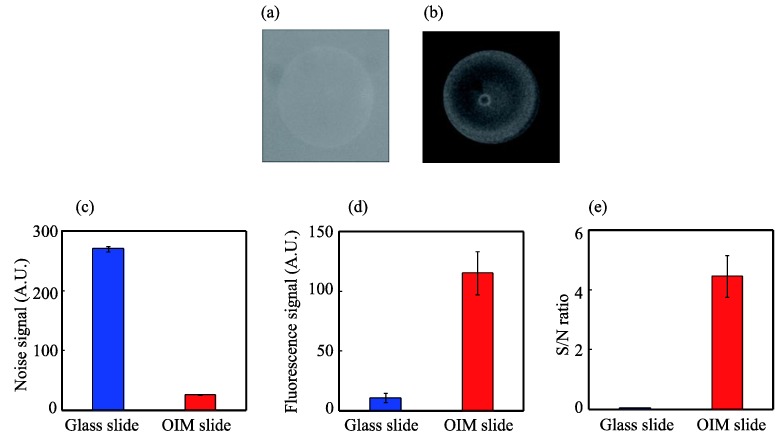
Fluorescence images of 100 ng/mL rhodamine B spotted on of the glass slide obtained with the general optical system (**a**) and the OIM slide obtained with the crossed Nicols optical system (**b**); analysis of the fluorescence images for the noise signal (**c**), fluorescence signal (**d**) and S/N ratio (**e**). The fluorescence signal is defined as the light intensity of a rhodamine B-spotted area subtracted by the background noise. The S/N ratio was calculated by dividing the fluorescence signal by the background noise. Each data point is the average of five spots. The error bars indicate the standard deviation.

The fluorescence image of the rhodamine B-spotted OIM slide was acquired with the crossed Nicols optical system to evaluate the contrast of the fluorescence image thereby obtained. For comparison, the fluorescence image of a rhodamine B-spotted bare glass slide was acquired using the general optical system. The fluorescence images obtained with the glass and OIM slides are shown in Figure 3a,b, respectively. Because the fluorescence with the glass slide was difficult to observe, the image contrast of the glass slide was adjusted using the image analysis software. A clearer fluorescence image was obtained from the rhodamine B spotted on the OIM slide than from that on the glass slide.

To analyze these images quantitatively, the background noise is defined as the light intensity of a rhodamine B unspotted area, which is shown in Figure 3c. The background noise with the OIM slide was found to be reduced and was approximately 1/10th that with the bare slide. The fluorescence signal is defined as the light intensity of a rhodamine B-spotted area subtracted by the background noise. An approximately 11-fold enhancement of fluorescence was obtained, as shown in Figure 3d. This rather low fluorescence enhancement is mainly due to the reduced excitation light intensity by the polarizer and the reduced fluorescence signal by the analyzer [19].

Because fluorescence image contrast is generally expressed in terms of the signal-to-noise (S/N) ratio, the S/N ratio was calculated by dividing the fluorescence signal by the background noise, as shown in Figure 3e. The S/N ratio was 0.04 and 4.4 for the glass slide and the OIM slide, respectively. Therefore, the S/N ratio with the OIM slide was increased by approximately 110-fold relative to that with the glass slide. This increase of the S/N ratio means that the image contrast with the OIM slide was improved up to 110-fold.

In our previously-reported results of high-contrast fluorescence imaging, a 100-fold increase of the S/N ratio had been achieved by a 1/8-fold reduction of the background noise and a 13-fold enhancement of the fluorescence signal [19]. Because the results presented in our previous report correspond well with the results obtained in the present study, the results of the present study are considered reasonable.

### 3.3. High-Contrast Fluorescence Microscopy for Biomolecular Analysis

**Figure 4:**
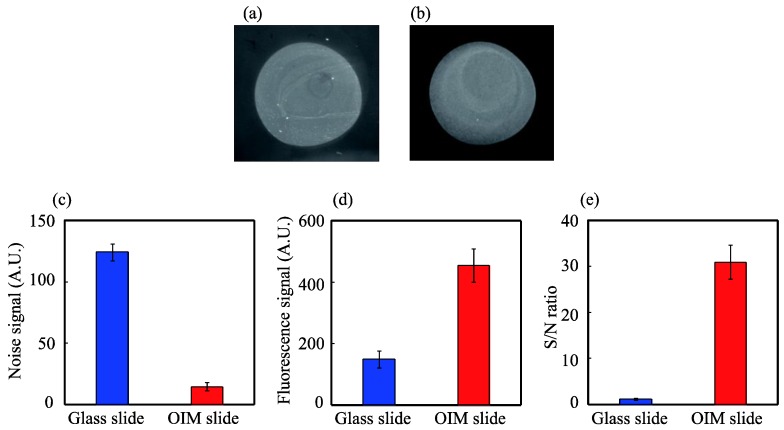
Fluorescence images of 1-µg/mL Cy3-labeled streptavidin detected with the glass slide obtained with the general optical system (**a**) and the OIM slide obtained with the crossed Nicols optical system (**b**); analysis of the fluorescence images for noise signal (**c**), fluorescence signal (**d**) and S/N ratio (**e**). The fluorescence signal is defined as the light intensity of a Cy-3 labeled streptavidin area subtracted by the background noise. The S/N ratio was calculated by dividing the fluorescence signal by the background noise. Each data point and the error bars indicate the average and the standard deviation, respectively.

Finally, Cy3-labeled streptavidin was detected using the biotin-immobilized OIM slide with the crossed Nicols optical system. For comparison, Cy3-labeled streptavidin was also detected using the biotin-immobilized bare glass slide with the general optical system. The obtained fluorescence images of the glass and OIM slides are shown in Figure 4a,b, respectively. To clearly observe the fluorescence image of the bare glass slide, the image contrast was adjusted using the image analysis software. Similar to the fluorescence image of rhodamine B, the fluorescence of Cy3-labeled streptavidin with the OIM slide can be observed more clearly than that with the glass slide.

To compare the S/N ratio quantitatively, the background noise and the fluorescence signal were analyzed, as shown in Figure 4c,d, respectively. An approximately 1/8-fold reduction of the background noise and three-fold enhancement of the fluorescence signal were achieved with the OIM slide. The S/N ratio was 1.3 and 3.1 for the glass slide and the OIM slide, respectively, as shown in Figure 4e. Therefore, the S/N ratio with the OIM slide was determined to be increased by approximately 24-fold, relative to that with the glass slide.

The S/N ratio for the detection of Cy3-labeled streptavidin was significantly lower than that for the detection of rhodamine B. The decreased fluorescence enhancement in this case is probably due to the non-specific adsorption of Cy3-labeled streptavidin to the slide unspotted area.

## 4. Conclusions

In this study, we demonstrated high-contrast fluorescence microscopy for biomolecular analysis based on polarization techniques using an OIM slide. When a linearly-polarized excitation light was used, the back-scattered excitation light from the OIM slide maintains the polarization of the incident light, whereas the fluorescence was unpolarized. On the basis of the difference between these polarizations, a crossed Nicols optical system composed of an analyzer rotated perpendicular to the polarization of the incident light was found to provide a maximum contrast for the fluorescence image.

To evaluate the image contrast with this crossed Nicols optical system, the fluorescence signal and the background noise from the rhodamine B-spotted OIM slide were measured. The image contrast with the OIM slide using the crossed optical system was improved up to 110-fold by an 11-fold enhancement of the fluorescence signal and a 1/10-fold reduction of the background noise compared with the glass slide using the general optical system. For the biomolecular analysis, a 24-fold improvement of the image contrast by a three-fold enhancement of the fluorescence signal and a 1/8-fold reduction of the background noise was achieved with the OIM slide, in comparison with the glass slide. The developed technique is expected to contribute to advancements in the field of bioimaging for subjects, such as molecules and cells.
